# The Frequency of Epidermal Growth Factor Receptor Mutation of Nonsmall Cell Lung Cancer according to the Underlying Pulmonary Diseases

**DOI:** 10.1155/2011/290132

**Published:** 2011-11-28

**Authors:** Kazuhiro Usui, Tomonori Ushijima, Yoshiaki Tanaka, Chiharu Tanai, Hiromichi Noda, Norifumi Abe, Hajime Horiuchi, Teruo Ishihara

**Affiliations:** ^1^Division of Respirology, NTT Medical Center Tokyo, 5-9-22 Higashigotanda Shinagawa, Tokyo 141-0022, Japan; ^2^Division of Diagnostic Pathology, NTT Medical Center Tokyo, 5-9-22 Higashigotanda Shinagawa, Tokyo 141-0022, Japan; ^3^Division of Chest Surgery, NTT Medical Center Tokyo, 5-9-22 Higashigotanda Shinagawa, Tokyo 141-0022, Japan

## Abstract

*Background*. Although epidermal growth factor receptor-tyrosine kinase inhibitors (EGFR-TKIs) are effective in patients with nonsmall cell lung cancer with epidermal growth factor receptor (EGFR) mutation, EGFR-TKIs have a risk of inducing fatal interstitial lung disease (ILD). The selection of chemotherapy based on the EGFR mutation status is recommended, however, the frequency of EGFR mutation in patients with ILD and the efficacy and safety of EGFR-TKI in patients with ILD and EGFR mutation are unknown. *Methods*. We retrospectively reviewed the association of the EGFR mutation status of nonsmall cell lung cancer and pulmonary diseases. Based on high-resolution computed tomography (HRCT) performed at diagnosis of lung cancer, patients were categorized into three groups: normal, emphysema, and fibrosis. *Results*. Of 198 patients with nonsmall cell lung cancer, we identified 52 (26.3%) patients with an EGFR mutation. EGFR mutations were identified in 43 (35.2%) of 122 patients with normal lungs, 8 (13.6%) of 59 with emphysema, and 1 (5.9%) of 17 with pulmonary fibrosis. Of the 52 patients with EGFR mutation, 43 patients received gefitinib. One patient with an EGFR mutation and fibrosis developed fatal ILD. There was not a significant difference in median overall survival from gefitinib treatment between never-smokers and smokers (797 days versus not reached; *P* = 0.96). *Conclusions*. Patients with sensitive EGFR mutation and normal lungs may benefit from an EGFR-TKI treatment even if they have smoking history.

## 1. Introduction

Gefitinib is a reversible epidermal growth factor receptor tyrosine kinase inhibitor (EGFR-TKI) used for the treatment of nonsmall cell lung cancer patients [1]. Although demographic and clinical factors such as East-Asian race, female gender, nonsmoking status, and adenocarcinoma were shown to be predictive of the efficacy of gefitinib, two pivotal studies showed that the presence of somatic mutations in the kinase domain of epidermal growth factor receptor (EGFR) strongly correlates with increased responsiveness to EGFR-TKIs in patients with nonsmall cell lung cancer [2, 3]. It was later found that the subgroups of patients with nonsmall cell lung cancer who had sensitivity to gefitinib had a high incidence of EGFR mutations [4, 5]. Selecting patients on the basis of EGFR mutations, rather than clinical factors, would likely result in a population with a greater sensitivity to gefitinib. First-line gefitinib for patients with advanced nonsmall cell lung cancer who are selected on the basis of EGFR mutations improves progression-free survival, with acceptable toxicity, compared with standard chemotherapy, although it failed to prolong overall survival [6, 7].

However, EGFR-TKI increases the risk of developing life-threatening interstitial lung diseases (ILDs). The estimated incidence of ILD is low in many countries (e.g., 0.3% in the United States) [8] but is relatively high (4 to 6%) in Japan [9, 10]. An older age, poor World Health Organization performance status, reduced normal lung area on computed tomography scans, preexisting chronic ILD, and concurrent cardiac diseases are known as risk factors for ILD in gefitinib treatment [10]. Although an assessment of pulmonary comorbidities, especially ILDs, is important to decrease the incidence of ILD induced by chemotherapy, the frequency of EGFR mutation in patients with pulmonary fibrosis and the clinical feature of these patients are not clear.

We reviewed 198 patients who were examined for EGFR mutation status and assessed the association of EGFR mutations with the underlying pulmonary diseases on chest high-resolution computed tomography (HRCT). 

## 2. Methods

The medical records of a series of consecutive patients with histologically- or cytologically-proven lung cancer, who were tested for EGFR mutation status in the Division of Diagnostic Pathology, NTT Medical Center Tokyo between April 2008 and November 2010, were retrospectively reviewed. The status of EGFR mutation was examined in a clinical practice, not investigational setting, to decide the indication of EGFR-TKI treatment. Although most patients with severe pulmonary fibrosis or squamous cell carcinoma were excluded from the EGFR mutation test in this period, gender, smoking status, and the existence of emphysema were not considered as the exclusion criteria of the test. Patients with emphysema and fibrosis on chest HRCT at the diagnosis of lung cancer were prospectively identified, and the data before lung cancer treatment was recorded to assess their risk of ILD. Only patients who had a chest HRCT scan, which was performed at diagnosis of lung cancer and was available for review, were included in the study. The study protocol was reviewed and approved by the Ethics Committee of NTT Medical Center Tokyo.

Patients were categorized into three groups; those with normal lungs (except for the tumor), emphysematous lungs, or fibrotic lungs, based on chest CT findings as described previously [11, 12]. Patients who met the following criteria were categorized as having emphysema: the presence of emphysema on CT, defined as well-demarcated areas of decreased attenuation in comparison with contiguous normal lung, and marginated by a very thin (<1 mm) wall or no wall, and/or multiple bullae (>1 cm) with upper zone predominance. Patients who met the following criteria were categorized as having fibrosis: the presence of diffuse parenchymal lung disease with significant pulmonary fibrosis on CT, defined as reticular opacities with peripheral and basal predominance, honeycombing, architectural distortion, and/or traction bronchiectasis or bronchiolectasis; focal ground-glass opacities and/or areas of alveolar condensation may be associated, but should not be prominent. Patients who met neither criterion emphysema nor fibrosis were categorized as normal. The electronic medical records were reviewed to obtain clinical and demographic data, including gender, age, smoking history, histology results, clinical stage of lung cancer, treatment, treatment-related toxicities, and survival.

### 2.1. EGFR Mutation Analysis

The presence of EGFR mutations was determined by the peptide nucleic acid-locked nucleic acid PCR clamp method as described previously [13]. The investigated EGFR-TKI sensitive mutations included G719C, G719S, G719A, L858R, L861Q, and exon 19 deletions, as well as a gefitinib-resistant mutation, T790M.

### 2.2. Statistical Analysis

Differences among the categorized groups were compared using either the two-sided chi-square test or Fisher's exact test. The survival was estimated by the Kaplan-Meier method, and differences in survival between the subgroups were analyzed by the log rank test. Data were analyzed using the StatView version 5.0J software package (Statistical Analysis Systems, Cary, NC, USA).

## 3. Results

### 3.1. Subtypes of EGFR Mutations

We examined the EGFR mutation status in 202 patients between April 2008 and November 2010. We excluded 4 patients from this study for the following reasons: one had small cell lung cancer, two had gastric cancer, and one had parotid cancer. Of the 198 patients with nonsmall cell lung cancer, 52 patients (26.3%) had EGFR-TKI-sensitive EGFR mutations, and one patient had an EGFR-TKI-resistant mutation (T790M) with an EGFR-TKI-sensitive mutation (Exon 19 deletion). The patient population in this analysis (Table 1) was a little young, including more female, less never-smoker, and less squamous cell carcinoma of the lung in comparison with the lung cancer cohort that we previously published [12]. 

### 3.2. The Variables Associated with the EGFR Mutation Status

We investigated the association of several variables with the EGFR mutations (Table 2). A two-sided chi-square test showed that gender (female), smoking status (never smoker), histology (adenocarcinoma), and chest CT findings (normal) were significantly associated with the presence of an EGFR mutation. Of 122 patients with normal lungs, 69 patients had no history of smoking and 53 patients had a history of smoking. The frequency of EGFR mutations (*n*, %) in patients with normal lungs did not differ between smokers (17, 32.1%) and never-smokers (26, 37.7%) (*P* = 0.5698).

### 3.3. Prognosis of Patients with EGFR Mutations Treated with Gefitinib

**Figure 1:**
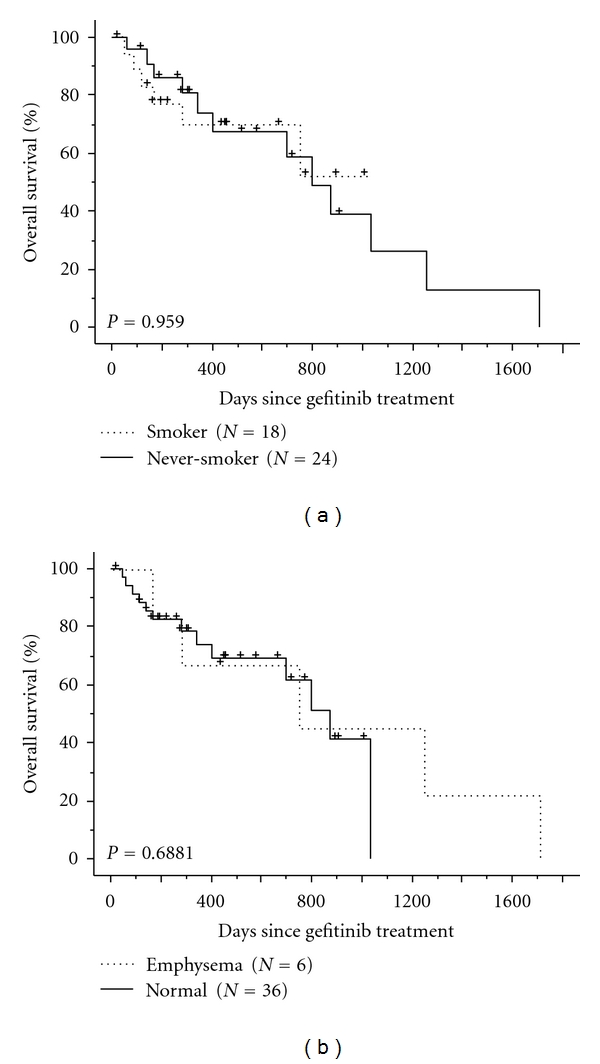
(a) Overall survival of patients with an EGFR mutation treated with gefitinib, according to smoking status (never smokers: solid line; smokers: dotted line). +: censored patient. (b) Overall survival of patients with an EGFR mutation treated with gefitinib, according to underlying pulmonary disease (normal: solid line; emphysema: dotted line). +: censored patient.

All patients with an EGFR mutation were treated in the Division of Respirology and Chest Surgery, NTT Medical Center Tokyo. Of the 52 patients with EGFR mutation, 43 patients received gefitinib. The clinical characteristics of the patients with an EGFR mutation treated with gefitinib are shown in Table 3. The median survival after gefitinib treatment was 797 days. We identified ILD in two patients during gefitinib treatment; one had no ILD before gefitinib treatment and one had pulmonary fibrosis. The patient with pulmonary fibrosis developed acute exacerbation of preexisting ILD on day 7 of gefitinib treatment and died on day 14 because of ILD. The survival curves of the 42 patients, excluding the patient with pulmonary fibrosis, according to smoking status and chest CT results, are shown in Figures 1(a) and 1(b), respectively. No differences in survival were observed between smokers (*n* = 18, MST not reached) and never-smokers (*n* = 24, MST 797 days) or between patients with normal lung (*n* = 36, MST 874 days) and those with emphysematous lungs (*n* = 6, MST 749 days) on chest CT. 

## 4. Discussion

We herein showed the frequency of EGFR mutation in nonsmall cell lung cancer to be high in patients with the following factors: female gender, no history of smoking, adenocarcinoma, and normal lungs on chest CT. A survival analysis of the patients with EGFR mutations, excluding one patient with pulmonary fibrosis, showed no differences between smokers and never-smokers or between patients with emphysema and those with normal lungs on chest CT.

There is considerable variability in the susceptibility of smokers to developing smoking-related pulmonary diseases [14–16]. The incidence of lung cancer is increased in patients with emphysema and fibrosis, and this effect is independent of the effect of cigarette smoking [17, 18]. We consider that smokers with emphysema or fibrosis are more susceptible to smoking-related inflammation compared to those with normal lungs. Although the frequency of EGFR mutation was low in patients with emphysema and fibrosis, the frequency in those with normal lungs was not different between smokers and never-smokers. Our data suggested that smokers with normal lungs were not susceptible to smoking-related inflammation, and that nonsmall cell lung cancer in smokers with normal lungs showed the same biological features to that in never-smokers. Further investigations are necessary to elucidate whether smoking-related pulmonary diseases and lung cancer might result from overlapping or associated genetic variants implicated in smoking-related inflammation.

Although a history of smoking and the coexistence of emphysema were negatively associated with the frequency of EGFR mutations, these clinical factors did not affect the prognosis of the patients with EGFR mutations treated with gefitinib. Toyooka et al. showed that epidermal growth factor receptor mutation, but not sex or smoking, is independently associated with a favorable prognosis of gefitinib-treated patients with lung adenocarcinoma [5]. EGFR-TKI treatment should be considered in patients with an EGFR mutation, even if they have a history of smoking or emphysema without fibrosis.

The presence of EGFR mutations in patients with pulmonary fibrosis was rare in this study. Only one (5.9%) of 17 patients with pulmonary fibrosis had an EGFR mutation. Preexisting chronic ILD is known as a risk factor for ILD in gefitinib treatment [10]. In this study, one patient with pulmonary fibrosis and an EGFR mutation treated with gefitinib developed fatal ILD. 

The present study had several limitations, including the fact that it was observational and uncontrolled in design and was performed at a single institution, with retrospective collection of data. The results may have been subject to some selection and treatment bias. The indications for therapy and the selection of treatment were not uniform for all patients, thereby limiting the evaluation of the effects of treatment. The data presented herein should not be interpreted as providing an appropriate evaluation of the efficacy of treatment, which will require randomized prospective studies. A multivariate analysis could not be performed due to the small sample size, and it was therefore not possible to evaluate the potential confounding effects of various other variables related to survival. However, the existence of emphysema and fibrosis on chest CT were prospectively identified at the diagnosis of lung cancer. The EGFR mutation status was identified before the EGFR-TKI treatment. Data on the demographic characteristics and survival of patients were unlikely to be affected by the study design.

In summary, the frequency of EGFR mutations in patients with normal lungs on chest CT was not different between smokers and never-smokers. Of patients with sensitive EGFR mutations and normal lungs on chest CT, smokers had a comparable prognosis with never-smokers. Selecting patients on the basis of chest CT, rather than the smoking status, would likely result in a population with a greater sensitivity to gefitinib. 

## Figures and Tables

**Table 1:** Patient characteristics NSCLC: nonsmall cell lung cancer: LCNEC; large cell neuroendocrine carcinoma.

Total number of patients	198
Age (median, range)	68, 28–92
Gender	
Female	86
Male	112
Smoking-status	
Never	74
Ex/Current	124
Histology	
Adenocarcinoma	169
Squamous cell carcinoma	9
Other NSCLC	15
LCNEC	4
Clinical stage of NSCLC	
IA	29
IB	14
IIA	2
IIB	6
IIIA	12
IIIB	30
IV	105
Chest CT	
Normal	122
Emphysema	59
Fibrosis	17
EGFR mutation	
Wild type	147
Ex18 G718S	1
Ex19 del	34
Ex21 L858R	15
EX19 del + Ex21 L858R	1
Ex 19del + T790M	1

**Table 2:** Patient characteristics and EGFR mutation status.

	Number	EGFR mutation (*n*, %)	*P*-value
Gender			
Male	112	17, 15.2%	*P* < 0.0001
Female	86	35, 40.7%	
Age			
<65	80	23, 28.8%	*P* = 0.5156
65≤	118	29, 24.6%	
Histology			
Adenocarcinoma	169	50, 29.6%	*P* = 0.0107
Nonadenocarcinoma	29	2, 6.9%	
Smoking status			
Never	74	29, 39.2%	*P* = 0.0139
Ex/Current	124	23, 18.5%	
Clinical stage of NSCLC			
I-IIIA	63	21, 33.3%	*P* = 0.1649
IIIB-IV	135	31, 22.9%	
Chest CT			
Normal	122	43, 35.2%	*P* = 0.0011
Emphysema	59	8, 13.6%	
Fibrosis	17	1, 5.8%	

**Table 3:** Characteristics of patients with an EGFR mutation treated with gefitinib.

Total number	43
Age (median, range)	67, 28–92
Gender	
Male	13
Female	30
Smoking-status	
Never	24
Ex/Current	19
Pack-years of smokers (median, range)	33, 2.5–225
Histology	
Adenocarcinoma	42
Squamous cell carcinoma	1
Clinical stage of NSCLC	
IB	2
IIIA	1
IIIB	7
IV	22
Recurrence	11
History of chemotherapy before gefitinib treatment	
No	28
Yes	15
EGFR mutation	
Ex18 G719C	1
Ex19 del	30
Ex21 L858R	10
Ex19 del + Ex21L858R	1
Ex19 del + Ex20 T790M	1
Chest CT	
Normal	36
Emphysema	6
Fibrosis	1
